# Alveolar morphology depends on ventilator settings: lessons from *in vivo *alveolar microscopy under static and dynamic conditions

**DOI:** 10.1186/cc9598

**Published:** 2011-03-11

**Authors:** D Schwenninger, S Schumann, J Guttmann

**Affiliations:** 1 University Medical Center Freiburg, Germany

## Introduction

In the context of lung-protective mechanical ventilation, knowledge about the global respiratory mechanics (for example, lung resistance and compliance) can be essential to guide the ventilatory therapy. From recent work it is known that the lung shows a significantly different mechanical behaviour when examined under static conditions (continuous ventilation interrupted by zero-flow or low-flow respiratory manoeuvres) compared with dynamic conditions (no interruption). However, the significance of this difference at the anatomical level of the alveoli has not yet been fully examined. This study aims to determine changes in morphology of subpleural alveoli under static and dynamic conditions in an animal model.

## Methods

A method for endoscopic intravital microscopy of lung tissue [1] was used to record videos of subpleural alveolar structures in a rat model. This specialized method allowed the continuously focused recording of the lung surface during any kind of respiratory manoeuvre, including continuous mechanical ventilation. Videos of alveolar structures were recorded during continuous mechanical ventilation (dynamic) at different levels of positive end-expiratory pressure (PEEP) and during low-flow manoeuvres (static) where the lung was slowly inflated up to an airway pressure of 40 mbar. Alveolar morphology was analysed using a dedicated semiautomatic image processing algorithm by tracking the change of area-size of the visible subpleural alveoli in the videos. The simultaneous change of area-size of different alveoli was averaged to get the mean alveolar area-size depending on the respective airway pressure. Comparison was done by calculating the difference of relative area-size increase in identical ranges of airway pressure under dynamic and static conditions.

## Results

Data from five animals mechanically ventilated at PEEP levels of 6 and 15 mbar showed a significantly smaller increase in area-size under dynamic compared with static conditions: 12% smaller at 6 mbar; 40% smaller at 15 mbar.

## Conclusions

Under dynamic conditions, the pressure-dependent change in alveolar morphology is significantly different compared with static conditions. We conclude that, to guide mechanical ventilation therapy, it is essential to determine respiratory mechanics under dynamic conditions.
